# Treatment of Cholera at Darlington

**Published:** 1832-10

**Authors:** 


### HOSPITAL REPORTS.

### TREATMENT OF CHOLERA AT DARLINGTON.

*Communicated by* Dr. Peacock.

*To the Editors of the London Medical and Physical Journal.*

Gentlemen: As many of the readers of your very respectable Journal may wish to know how the prevailing epidemic is successfully treated, I have sent you the practice of Dr. Macfarlan (my worthy partner,) and myself.

The cholera touched at Darlington on the 9th of last April. We had only one patient at that time (a T. Gales), whom we treated according to the Indian method, with large doses of opium and calomel, which did not succeed. The disease returned here more briskly in the autumn: at this time the English cholera prevailed very much in Darlington; but we do not consider any case of the *Asiatic* species that is not attended with pale, watery discharges, and the frame cold and collapsed. The effects of medicine was so much the same in every case, that there will only be occasion to describe the effects of it in a very few, to understand it thoroughly.

Mrs. Chilcott, of Church street, aged fifty-six, was attacked with violent retchings and looseness, August the 8th, for which her good neighbours poured in their usual nostrums, and other *comfortable things.* She struggled on in this way during a day and a half, when, observing her turning blue in her face and hands, they sent for assistance. Her thirst was great, her pulse weak and difficult to count, her limbs suffered under violent spasms, and she was cold as a corpse. Dr. Macfarlan ordered her a grain of calomel, to be repeated every fifteen minutes, washing it down with a tablespoonful of a mild saline mixture. Her stools, and what she vomited, had no resemblance to natural discharges. In a little time she felt tired of the mixture; so she was ordered to drop it, and go on with the calomel, which she continued every quarter of an hour during the next night, with very evident advantage. The dark blue stain on her face and hands began to be less conspicuous; her pulse became fuller and softer; her spasms had ceased; and altogether she expressed herself more comfortable, and confident in her recovery. It should be remarked, that this woman was soon acted upon by mercury, and at the end of the third day she detected the drug in her mouth; but she yet had copious evacuations by stool of various colours, but more of the natural odour. She had now to contend with the consecutive fever, which was rather aggravated by the sore mouth, but soon got well.

On August the 8th, Miss D. B. had a very severe attack: she began in the evening, but did not call upon us till midnight, when she was considered in great and immediate danger; in short, in a state of complete collapse. Besides her face and body being shrunk, there was a dark blue stain upon her hands. She began with a grain of calomel, which was repeated every fifteen minutes, with a tablespoonful of a mild saline mixture every hour, which was continued through the next day. About nine in the morning, the retchings began to abate, but the stools did not change before night. She had taken fifty-one of the mercurial pills before the consciousness of approaching recovery came upon her, but never had any soreness of the mouth.

I will just give another case before I close my relation of the means employed. We have had upwards of twenty-three cases of complete collapse, besides these three, in which we have not lost one patient; and, when called upon, we have such a confidence in the means we use, that we go to work in perfect security.

The next case is that of Mrs. Bell, aged sixty, the wife of an innkeeper between Darlington and Croft, a lusty woman of sober habits, who was attacked, August 25th, at one in the morning, but had the hardihood to struggle with it till eleven A.M. I found the vomiting and purging of a colourless fluid very copious, with an intense pain about the scrobiculus cordis; she was very cold, with a clammy sweat over the whole body, but no cramps or spasms; and I trust we evaded the blue tinge; her pulse was 130, but very weak and thready. I gave her immediately a grain of calomel, and told her to repeat it every ten minutes, with a tablespoonful of the following mixture every half-hour: ℞. Supertart. Potassæ et Sodæ ℥i.; Aq. font. ℥x. M. fiat mistura. I saw her again at five P.M., when her skin was warmer, and had lost its clamminess; the pain at the stomach was much abated; the pulse now only ninety, and much fuller and softer; the retching had in some measure abated. I had seen so much of the complaint during the last month, that I now told my patient "I made sure of her." The medicines were continued during the night, at somewhat longer intervals; and the next morning her stools were very black and somewhat feculent, and all went on well. I remain, &c.

J. PEACOCK, M.D.

P.S. I have been told that Dr. AYRE, of Hull, has been very successful with small doses of calomel and a little opium: we do not use an atom of opium or of brandy in any stage of the cholera.

*Darlington, co. Durham; Sept.* 16*th*, 1832.

